# AI in drug design: evolution or revolution?

**DOI:** 10.1042/ETLS20240005

**Published:** 2025-06-17

**Authors:** Darren V. S. Green

**Affiliations:** DesignPlus Cheminformatics Consultants Ltd, Hertfordshire, U.K

**Keywords:** cheminformatics, computational models, generative design, machine learning, protein design

## Introduction

The pharmaceutical industry is familiar with the ‘hype cycle of technologies, artificial intelligence (AI) being the most recent. AI is best thought of as a nested set of capabilities: machine learning (ML; models that learn from legacy data), deep learning (ML models that mimic human brain processes), generative AI (use of ML to create original content) and the ultimate goal of artificial general intelligence (systems capable of conducting scientific research and discovering new knowledge).

Great claims are made for AI in drug discovery – a revolution is coming according to McKinsey [1]. There has been large amounts of backing for AI-based startups, with an estimated $4 billion invested between 2018 and 2022 in the leading 20 companies [2] and the size of the AI services market in drug discovery expected to reach almost $8 billion per annum by 2030 [3]. Recently, Xaira Therapeutics spun out of the University of Washington Baker lab with $1 billion in funding [4]. Given the lack of impact on pharma productivity from previous technology ‘game-changers’ [5], how much is based on real evidence and how much is wishful thinking? Exactly where and how will AI disrupt established practices in drug discovery? This perspective aims to shed some light on these questions and will hopefully convince you that there is already enough evidence that, this time, the journey along the technology hype curve will be different.

### AI methods

Without descending into the nuances of deep learning network architectures and such like, it will be useful to introduce common ML terminology and utility. The comprehensive review by Yang et al. [6] is recommended for further reading.

‘Classical machine learning’ is a term generally applied to the collection of methods which pre-date ‘deep learning’. Supervised learning methods (i.e. those which are trained to predict a specific labelled end-point such as logP) include support vector machines, naïve Bayes and random forests. Unsupervised learning methods (i.e. where the data are unlabelled) include clustering, k-nearest neighbours, principal component analysis and self-organising maps. These methods are fed descriptors (e.g. chemical structure fingerprints [7]) and produce a mathematical model that relates the descriptors to the desired endpoint (supervised) or allows a data-driven representation of the molecules in the descriptor space (unsupervised).

Deep learning methods have been key to the emergence of modern AI. Deep learning typically refers to a learning system incorporating multiple layers of artificial neural networks. Such networks are very flexible learners and are able to model many types of data (e.g. medical images, face recognition, speech, music and, of course, molecular data) and highly complex, non-linear relationships. They are particularly powerful when given very large data sets, for example the 1.2 million images used by the breakthrough AlexNet image classification system [8].

A key departure from classical ML is the ability of deep learning models to learn the most effective representation of the data, rather than use fixed, human-engineered descriptors. Molecules can be represented as graphs or as SMILES strings [9] or proteins as sequences of their shorthand amino-acid letters, with their actual representation in the model refined by the model training process.

The flexibility of deep learning networks has enabled a large number of variants and types of learning:

- Multitask learning allows learning of several, related endpoints (e.g. IC50 data from kinase panels) in parallel, making use of a shared representation which can be very useful where some endpoints have large data and others small data.
- Transfer learning enables fine-tuning of a model which has been pre-trained on a large corpus of data, by further training on a much smaller but focussed data set.
- Reinforcement learning is a reward-driven learning strategy that enables the optimisation of a model to predict an outcome without a priori knowing how to optimise; it is often used in combination with other computational models which may impose penalties (e.g. developability models) or rewards (e.g. fit to a pharmacophore model).
- Contrastive learning is a semi-supervised learning method which attempts to learn a latent space where similar data are close and dissimilar data are far apart. It is particularly useful at integrating disparate data types such as image and chemical structure data [10].
- Diffusion models [11] are a class of supervised learning methods gaining in popularity. Noise is incrementally added to the training data (e.g. a set of images) until the new data set is a Gaussian distribution. A deep learning model is then trained to be able to follow the reverse process (i.e. start with noise and recreate the input image). This creates a model that is a very efficient generative tool for images and, increasingly, molecular design [12,13]
- Recurrent neural networks models (RNN) have proved a powerful tool for generative chemistry [14]. Designed for modelling time-series and sequence data and particularly useful for language, translation and speech models, RNNs (particularly the long short-term memory variant [15]) use the context of a word or character to modify the prediction for what the next word or character will be.
- Large language models (LLMs) [16], a hot topic due to the extraordinary impact of OpenAI’s Chat GPT and similar models. These are extremely large models, trained by extremely large data sets. In contrast with RNNs, LLMs use a transformer architecture that enables self-learning plus parallelisation of training. LLMs are able to understand entire conversations and context. Interestingly, the feature of LLMs that often irritates everyday use (hallucination) is the one that is most useful for molecule design – producing plausible SMILES or protein sequences that have never been reported.

One other learning technique should be mentioned. Active learning is an optimisation method that uses model uncertainty to guide the next data acquisition, either from an existing data set or from the next experiment in a design–make–test cycle. Generally, active learning approaches will seek to suggest data that will improve the model (‘Explore’), until the model has reached a point where it can confidently predict (‘Exploit’).

### Application of AI in small molecule discovery

ML in chemistry is not new. In fact, chemistry has its own name for statistical models: quantitative structure activity relationship (QSAR) models. Initially, these were linear regression models, the first being published in the 19th century(!) by Overton [17] and Meyer [18]. These ideas were famously developed by Hansch & Fujita [19]. QSAR has continued to evolve as new methods were invented [20], the chemistry community popularising the multivariate technique of partial least squares [21]. QSAR modellers were early adopters of neural networks [22], kernel ML methods [23], random forests [24], active learning [25], automated design [26], AI-based design processes [27], Pareto-based multi objective designs [28,29] and automated QSAR modelling/MLOps [30,31]. QSAR models have been used in the design of marketed drugs [32] and are established tools in a regulatory setting for risk assessments of organic compounds [33].

If ML is not new to drug design, why then the current, excited, interest and what has enabled it? The growth in computing power (an iPhone 12 is *5000* x faster than the Cray-2, the world’s fastest supercomputer from 1985!), and almost commodity pricing of very large memory and storage has enabled computational scientists to employ methods that were hitherto either impractical or infeasible. On a practical level, great computational power has also accelerated the speed with which researchers develop new solutions, reducing the iteration time for each cycle of testing. Here is a non-exhaustive list of the most interesting developments (note: not all are AI applications):

- Large-scale, big data cheminformatics, such as matched molecular pairs [34,35] and series [36]
- Large-scale ML hyperparameter optimisation to yield optimal models [37]
- Large enumerations of readily accessible chemistry space [38]
- Free energy perturbation (FEP, invented in 1987 and now usable!) [39]
- Deep learning QSAR models for large datasets [40]
- Deep learning-based generative chemistry [41]
- ML-based forcefields with DFT levels of accuracy [42]
- Accurate protein structure prediction with Alphafold & RosettaFold [43,44]
- Multi-task [45] and multi-modal [46] modelling of complex data sets
- Small [47] or large language models trained on chemistry [48] and protein sequence [49]
- Transfer learning from pre-trained/foundational models for small data sets [50,51]
- Federated modelling for safe data sharing between companies [52,53]

This is an impressive list of capabilities, but do they work in the real world? In short, it appears so. In their review of generative chemistry, Du et al. [41] cite no fewer than 37 published examples of laboratory validated small molecule design using generative chemistry methods.

**Figure 1:**
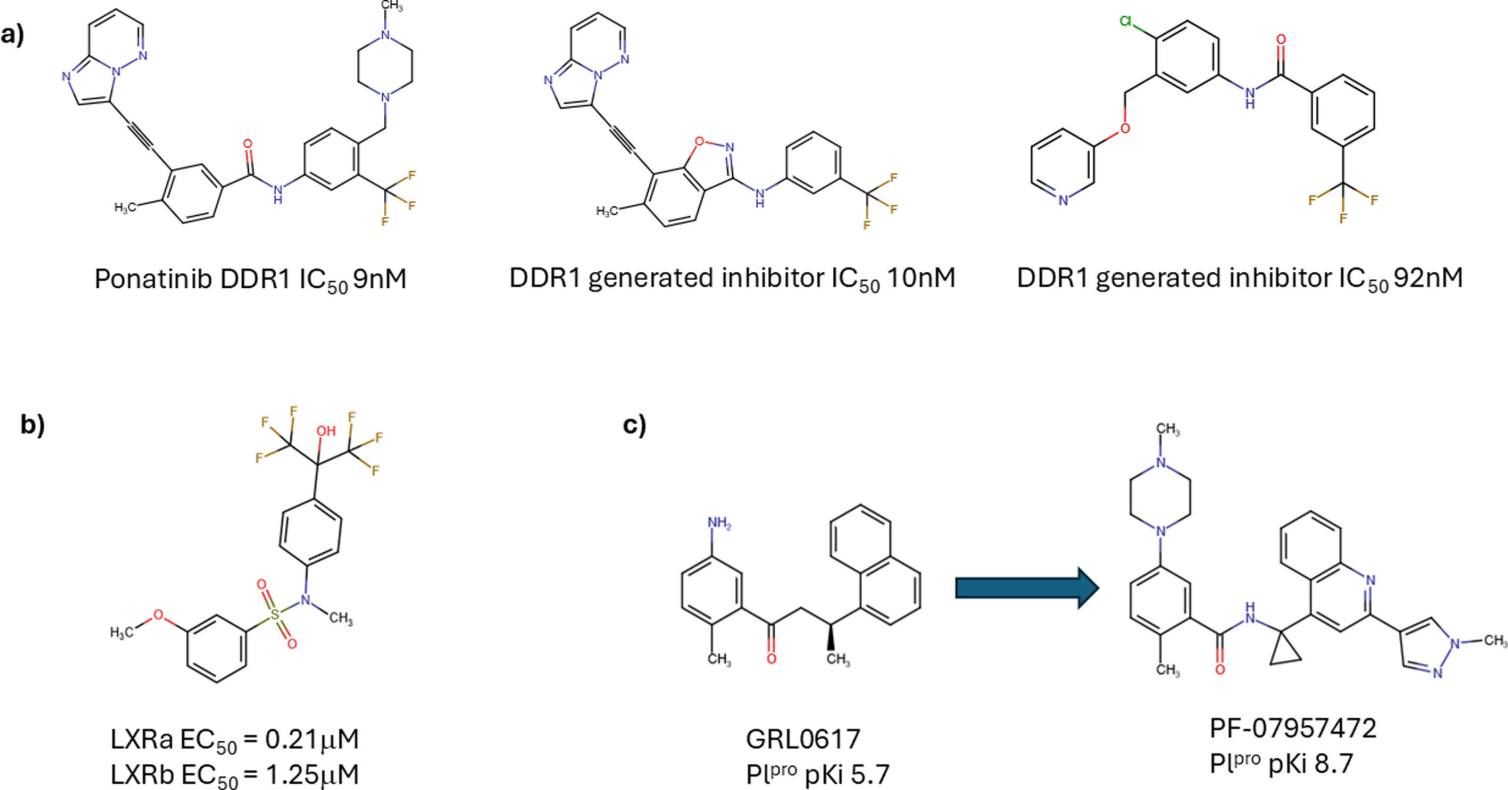
Chemical structures of compounds designed using generative chemistry methods. (**a**) DDR1 inhibitors: the marketed drug Ponatinib and those designed from reference [54] and reference [55].(**b**) An LXR ligand design in reference [56]. (**c**) The starting (GRL0617) point and optimised (PF-07957472) compound designed in reference [57].

The first published example of generative chemistry design is that of Insilico Medicine’s DDR-1 inhibitor [54], designed, synthesised and tested in 21 days. This was a controversial example, being extremely close to a known marketed drug Ponatinib (Figure 1a) and subject to a ‘well any chemist would have done that’ response. A more charitable view needs to be taken – these new design paradigms must be able to do the ordinary as well as – hopefully – the extraordinary. A more novel DDR-1 inhibitor was discovered by Yoshimori et al. [55] (Figure 1a) by coupling a generative chemistry model with a traditional pharmacophore approach. More ambitious was the coupling of an automated design system with an automated on-chip chemical synthesis platform to generate novel LXRa agonists (Figure 1b) [56]. More recently, a collaboration between Pfizer and PostEra reported the ML-driven discovery of a series of potent, selective and orally available SARS-CoV-2 PLpro inhibitors, with the lead compound (active in a mouse model) identified in less than eight months [57] (Figure 1c).

There are other validated computational protocols for automated design that use more traditional computational chemistry and cheminformatics. The first published example of modern automated design was provided by Besnard et al. [58], whereby novel compounds were generated using cheminformatics methods and scored with QSAR models which were combined to drive multi-objective optimisation. Using this approach, CNS-penetrant, selective dopamine D2 inverse agonists and compounds fitting a polypharmacological profile were designed. Schrödinger has pioneered large-scale cheminformatics and free energy simulation to drive lead optimisation. The discovery of the Malt-1 inhibitor SGR-1505 [59] used a computational pipeline involving the generation of 8 billion compounds through reaction-based enumeration, an Active Learning FEP protocol to generate a machine model that could triage large numbers of compounds before committing to full free energy simulation, followed by multiparameter optimisation using ML QSAR models. By using this intense computational process, the project needed only 10 months and 78 compounds synthesised to optimise to a clinical candidate [60].

ML has been applied to hit identification or virtual screening. The size of available ‘make to order’ libraries is becoming extremely large – over 10^12^ compounds and growing – and searching them with traditional methods (pharmacophore searching, docking) is accordingly expensive. Klarich et al. [61] utilised an active learning approach called Thompson sampling to make the search process more efficient, needing to evaluate only 1% of the virtual library to find >50% of the known hits. The approach can be coupled with any type of screening method; they demonstrate 3D shape searching and docking. An alternative solution to this problem is the NGT (NeuralGenThesis) methods of Oliveira et al. [62]. NGT uses deep learning to project a 3 trillion compound vendor catalogue into a ‘latent space’ which has an associated decoder to regenerate chemical structures. The virtual screen can then iteratively sample promising compounds from the latent space, generate the structures via the decoder, and score them using, in this case, docking to a crystal structure of the activated receptor, an AlphaFold model and a homology model. The example given describes the identification of potent and selective inhibitors of the melanocortin-2 receptor.

More ambitious than searching in pre-defined chemical libraries is the *de novo* generation of hit molecules. Thomas et al. [63] utilised an LLM pre-trained on ChEMBL [64] with the goal of generating novel chemical structures with a low-energy docking score for seven known A2A protein crystal structures, alongside a variety of developability metrics such as logP, hydrogen bond donors and rotatable bonds. After extensive filtering, nine compounds were synthesised, yielding three nanomolar ligands with confirmed functional activity, two of which are novel chemotypes.

An emerging hit discovery strategy is to apply ML to screening data from DNA-encoded libraries and use the resulting model to predict activity in databases of commercially available compounds, thus saving the resource cost of off-DNA resynthesis. An example of this is the discovery of a low micromolar, first-in-class ligand for WDR91 [65], testing only 150 commercial compounds.

### Biologics

Protein design is a younger discipline than its small molecule cousin [66]. It has its origins in protein engineering, where known proteins are mutated to gain information, to optimise a function, or repurpose the protein for another function. In this use case, the protein structure fold, stability and dynamics tend to be retained. This is not a trivial pursuit, demonstrated by the award of a Nobel Prize in 2018 [67]. In the last two decades, however, protein design has made extraordinary progress utilising both ‘physics-based’ structural modelling and of course Machine Learning [68], culminating in the award of its own Nobel Prize in 2024 [69]. AlphaFold [70], RosettaFold [44] and the evolutionary-scale LLM (ESM) family [71] are leading examples of these impressive new capabilities that are set to affect the design of enzymes, antibodies, vaccines, nanomachines and more [68]. These methods are built on the billions of publicly available sequences which sample diverse protein families and encode evolutionary constraints on the sequence–structure relationship. This is supplemented by >200,000 protein structures in the PDB [72].

**Figure 2:**
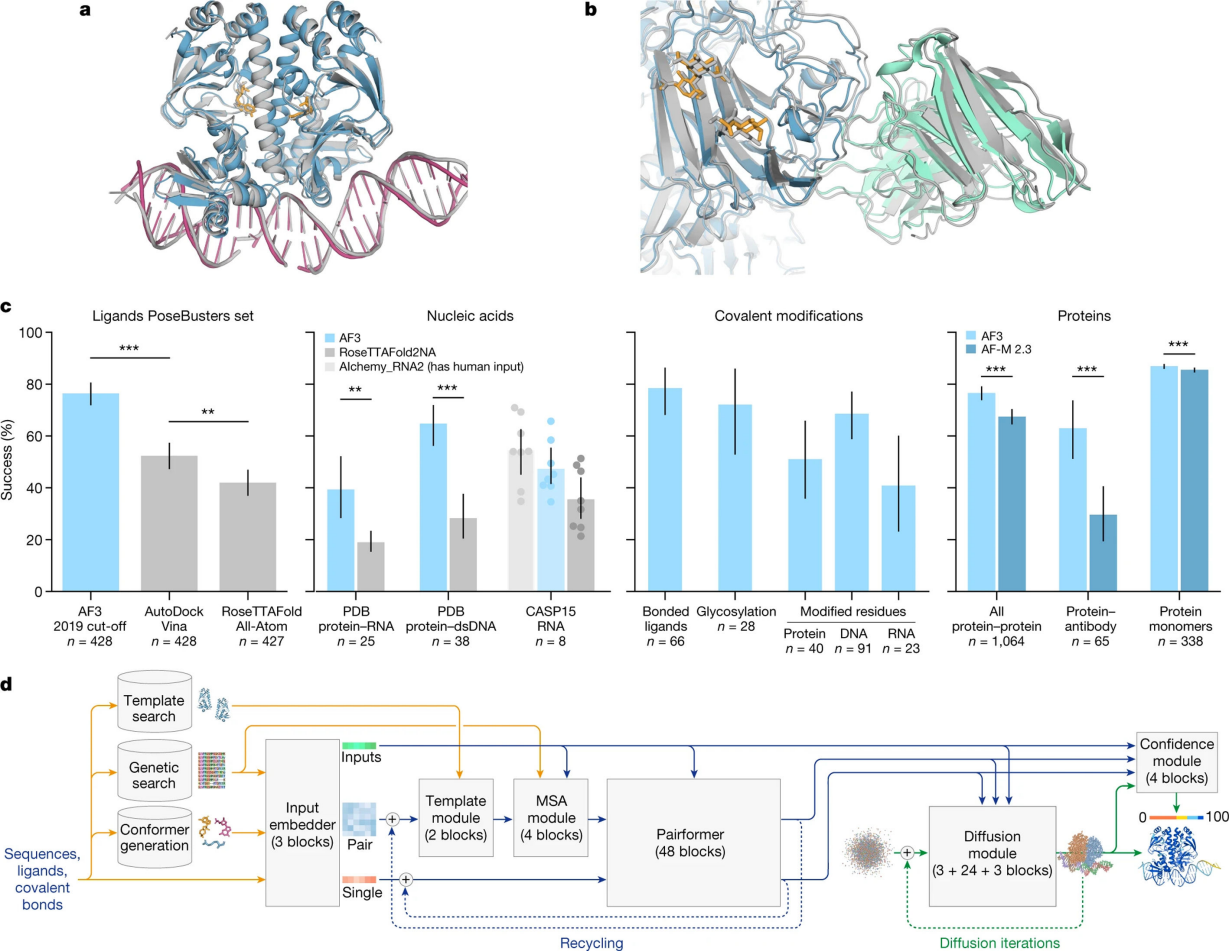
Example structures predicted using AF3. (**a**) Bacterial CRP/FNR family transcriptional regulator protein bound to DNA and cGMP (PDB 7PZB). (**b**) Human coronavirus OC43 spike protein, 4665 residues, heavily glycosylated and bound by neutralising antibodies (PDB 7PNM). (**c**) AF3 performance on PoseBusters (v.1, August 2023 release), a recent PDB evaluation set and CASP15 RNA.(**d**) AF3 architecture for inference. The rectangles represent processing modules and the arrows show the data flow. Yellow, input data; blue, abstract network activations; green, output data. The coloured balls represent physical atom co-ordinates. Reproduced from reference (70) under the Creative Commons Attribution 4.0 International License (https://creativecommons.org/licenses/by/4.0/)

AlphaFold successfully bridged the disciplines of bioinformatics, structural biology and ML by using multiple sequence alignments (MSA), patterns of conformations/interactions observed in protein crystal structures, and a deep learning architecture adopted from natural language processing [73]. AlphaFold3 was trained to predict not only protein structures but also biomolecular complexes of proteins, nucleic acids and their ligands (Figure 2). AlphaFold3 has an updated learning architecture to reduce dependency on the MSA and has introduced a diffusion model that creates the atomic co-ordinates of the models.

RosettaFold builds on its protein-modelling heritage, utilising a residue-based presentation of amino acids and DNA bases, 1D sequences, 2D pairwise distance information from homologous proteins and 3D co-ordinate information as input to a deep learning architecture. The RoseTTAFold Diffusion method (RFDiffusion) [74] utilises a Diffusion Model to create the final atomic model.

The ESM family of models starts from a completely different area of ML – that of LLMs. ESM-2 is trained using over 65 million unique sequences, using a technique known as masked language modelling, whereby sequences in the training set have (in this case) a random 15% of amino acids ‘masked’, and the model is trained to predict them correctly. This strategy removes the need for sequence alignments. The sequence model is then passed to a folding model which benefits from a low-resolution picture of protein structure (such as residue-residue contact probabilities) that has been learnt by the LLM.

Successful applications of state-of-the-art protein design tools are impressive. The AlphaProteo design system [75] (based on AlphaFold) designed novel protein binders for eight diverse target proteins. Binders were experimentally verified for seven proteins, with affinities ranging from 80 pico-molar to low nano-molar. Two were tested for biological function, demonstrating inhibition of VEGF signalling in human cells and SARS-CoV-2 neutralisation in Vero monkey cells. Designed binder and binder-target complex structures were confirmed with X-ray crystallography and Cryo-EM.

RFDiffusion was able to design de novo protein binders for four protein targets: Influenza Haemagglutinin A, IL-7 Receptor-α, PD-L1 and TrkA receptor with Kd of 28 nM, 30 nM, 1.4 mM and 328 nM, respectively. In the same paper, *de novo* proteins with mixed alpha-beta topologies are designed, characterised with circular dichroism and their thermostability validated. Symmetric oligomers with unprecedented structures were designed, as were novel proteins designed to ‘scaffold’ known binding sites (e.g. the scaffolding of the p53 helix that binds MDM2) and enzyme active sites (e.g. a retroaldolase active site triad TYR1051-LYS1083-TYR1180).

The ESM LLM was used to affinity mature seven human immunoglobulin G (IgG) antibodies that bind to antigens from coronavirus, ebolavirus and influenza A virus representing diverse degrees of maturity. In each case, affinity was improved after creating 20 or fewer new variants of each antibody, across only two rounds of evolution. Although many of the suggested mutations would be considered common in nature, 5/32 affinity-enhancing mutations involved a rare or uncommon substitution. One surprising but effective substitution was that of a glycine in the wild-type (observed in 99% of natural antibody sequences) to a proline (observed in <1% of natural sequences).

‘One shot’ ML enabled *de novo* antibody design has been reported [76] using a model trained on known antibody-antigen complex structures. As validation of the method, the known product trastuzumab and its antigen HER2 were taken as a case study. Novel HCDR3 and HCDR123 sequences (diverse with respect to trastuzumab and each other) were generated from the model, which were validated using SPR, with 71 having affinities less than 10 nM. Three antibodies had a higher affinity for HER2 than trastuzumab.

### LLMs as an orchestrator of experiments

**Figure 3:**
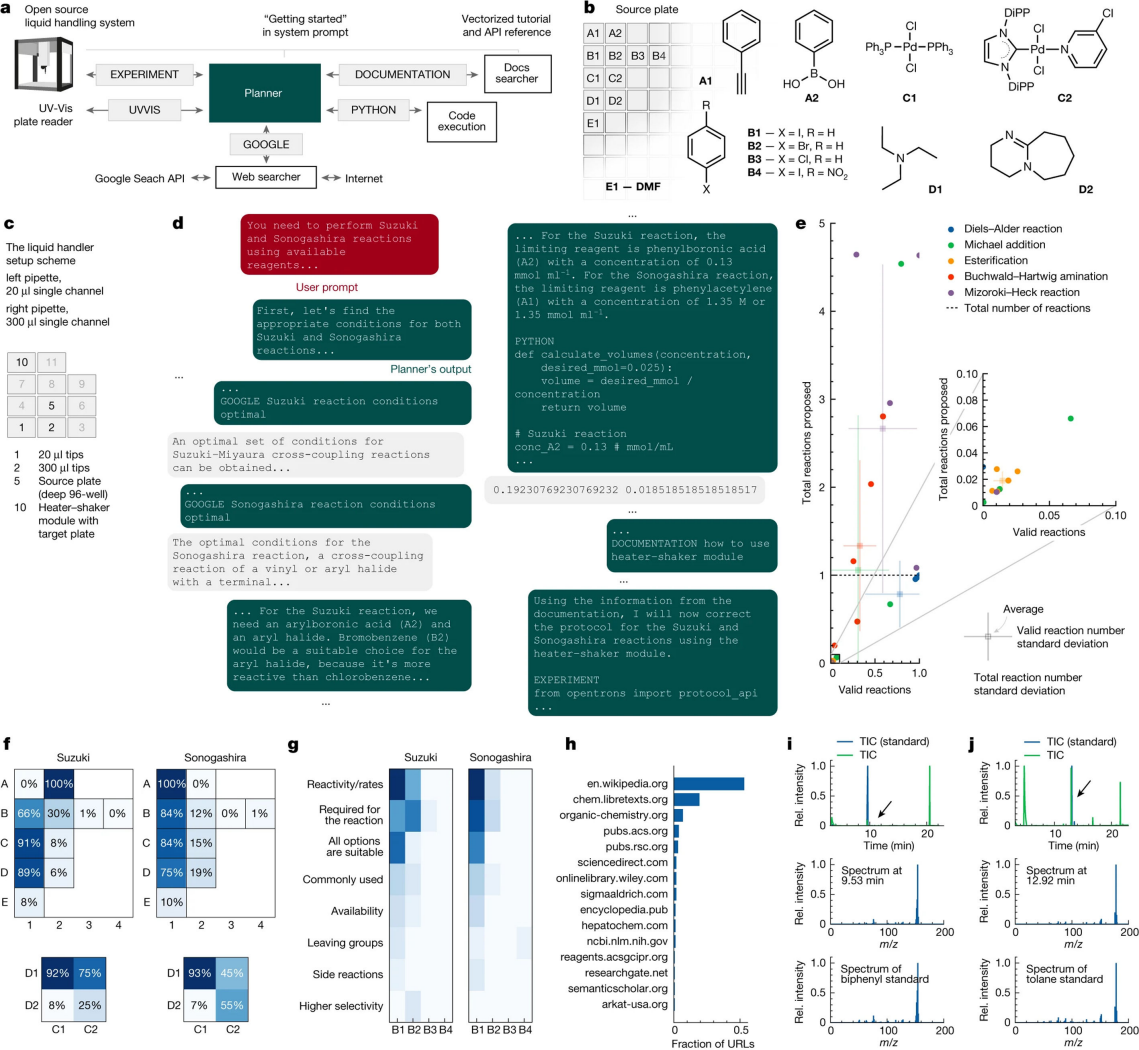
Cross-coupling Suzuki and Sonogashira reaction experiments designed and performed by Coscientist. (**a**) Overview of Coscientist’s configuration. (**b**) Available compounds (DMF, dimethylformamide; DiPP, 2,6-diisopropylphenyl). (**c**) Liquid handler setup. (**d**) Solving the synthesis problem. (**e**) Comparison of reagent selection performance with a large dataset. (**f**) Comparison of reagent choices across multiple runs. (**g**) Overview of justifications made when selecting various aryl halides. (**h**) Frequency of visited URLs. (**I and j**) analytical data on the synthesised materials compared with pure standards. Reproduced from reference [78] under the Creative Commons Attribution 4.0 International License (https://creativecommons.org/licenses/by/4.0/)

No article would be complete without mentioning the integration of ML with experiment planning and execution. ChemCrow [77] and Coscientist [78] are LLM-based systems which design, plan and execute complex experiments. The user interface is the LLM and it is augmented with modules or agents which are designed for very specific tasks (e.g. web search, retrosynthetic analysis, structure to price, programming of liquid handlers). The LLM is able to take user instruction, e.g. ‘Find and synthesize a thiourea organocatalyst which accelerates a Diels-Alder reaction’, orchestrate the various tools to produce an answer and even create code to drive an automated synthesis platform. ChemCrow was able to design a new chromophore with a predicted maximum absorption wavelength of 369 nm and a two-step synthetic protocol from available starting materials. Coscientist was able to orchestrate iterative experiments to optimise conditions for both Suzuki coupling and Buchwald–Hartwig reactions (Figure 3).

### Are we there yet?

The above examples illustrate the potential that ML tools have to improve the rate of scientific discovery. However, these examples represent the state of the art, and publication bias (see later) is very real – these are published because they are successful. Perhaps, the best way to comment is to explore the limitations of the current tools.

First and foremost, ML relies on good data and preferably in large quantities. Where this exists, the resultant models can be impressive. But large high-quality scientific data are expensive to acquire: the cost of replacing the protein structure data in the PDB is conservatively estimated at $20 billion [79]. Data are the recurring issue, particularly for applications such as drug discovery where the application domain is always outside or on the edge of the training set [80]. Extrapolation is the requirement for useable ML models, and here, we are still struggling to understand what it is these models are actually learning. High-profile docking models were exposed as learning the data but no physics [81], whilst there are justifiable concerns on overfitting to errors in data [82]. Even AlphaFold3 has been shown to memorise conformations and not the physics which underpin them [83]. This potential for memorisation and lack of causal reasoning has led to a call to make AI be more scientific [84]. As one researcher noted [85], LLMs and other AI systems ‘lack the basic capacities for intersubjectivity, semantics and ontology that are preconditions for the kind of collaborative world-making that allows scientists to theorize, understand, innovate and discover’.

ML researchers rely on public domain benchmarks to judge the effectiveness of their new algorithms. It was the CASP (Critical Assessment of Structure Prediction) [86] challenges that enabled the revolution in protein structure prediction. There are no comparable benchmarks for real world drug discovery, and this remains a constraint on the field [87]. The literature is full of publication bias – there is no ‘Journal of Failed Chemical Reactions’.

### Evolution or revolution in drug design?

There is no ignoring the impact of ML or denying the potential impact in the coming years. How will it benefit drug discovery? It will depend on the implementation because this is a disruptive technology – to get the best out of it requires business process re-engineering [88]. AI demands data. With the appropriate data, the ML drive design and discovery will perform well. Getting the right data quickly and cheaply is the challenge.

Biologics discovery will most probably be first to feel the benefits as much of the necessary experiments are largely automated, and the performance of the foundational models is impressive.

In small molecule discovery, we are likely to see a dual track adoption. On one hand, new companies are built around automated design (e.g. ExScientia, now merged with Recursion) in much the same way that companies were formed to pursue Structure Based Design (Vertex) and Fragment Based Design (Astex). More established companies will need to overcome the well-established “human centric” model [89] of the designer-maker medicinal chemist, which is not well placed to adopt the new approaches. Change management in this community can be a difficult business [90]. Indeed, McKinsey estimates that change management costs are three times the development of generative AI solutions [91]. But change will need to come, and it is not out of place to mention Kodak [92] as a cautionary tale at this point.

Moving ML models away from interpolation and towards extrapolation/reasoning and mechanistic thinking is necessary. AlphaFold3 has probably extracted as much out of current public domain data as is possible. A possible solution to both of these issues could be greater integration of physics-based models and simulation as a source of data [93].

What we do know is that the pace of change in the ML world is faster than any previous technology change we have witnessed. The next few years will see the growth in ‘lab in the loop’ [94,95] and even Autonomous Discovery [96–98] approaches as ML, informatics and experimental automation converge. We will remember this period as the time when a Revolution started. Drug design will look very different in the future, even if at the moment it is difficult to predict what the end state will look like.

**Summary Points**

- Machine learning (ML) in drug discovery builds on decades of innovation in bioinformatics, cheminformatics and computational chemistry.
- ML adds significant capabilities to the computational toolbox, in some cases providing a significant leap in performance.
- The literature is full of successful examples of ML-driven design in both small molecules and biologics.
- Having the right data – both quality and quantity – is key to success.
- This is a disruptive technology which will change how scientists work.

## Abbreviations

AI: artificial intelligence
FEP: free energy perturbation
LLM: large language model
ML: machine learning
QSAR: quantitative structure activity relationship
RNN: recurrent neural network
